# Defining the nuanced nature of redox biology in post-traumatic stress disorder

**DOI:** 10.3389/fphys.2023.1130861

**Published:** 2023-03-16

**Authors:** Emily C. Reed, Adam J. Case

**Affiliations:** ^1^ Department of Psychiatry and Behavioral Sciences, Texas A&M University, Bryan, TX, United States; ^2^ Department of Medical Physiology, Texas A&M University, Bryan, TX, United States

**Keywords:** posttraumatic stress disorder (PTSD), psychopathy, trauma, free radicals, oxidant, antioxidant

## Abstract

Post-traumatic stress disorder (PTSD) is a mental health disorder that arises after experiencing or witnessing a traumatic event. Despite affecting around 7% of the population, there are currently no definitive biological signatures or biomarkers used in the diagnosis of PTSD. Thus, the search for clinically relevant and reproducible biomarkers has been a major focus of the field. With significant advances of large-scale multi-omic studies that include genomic, proteomic, and metabolomic data, promising findings have been made, but the field still has fallen short. Amongst the possible biomarkers examined, one area is often overlooked, understudied, or inappropriately investigated: the field of redox biology. Redox molecules are free radical and/or reactive species that are generated as a consequence of the necessity of electron movement for life. These reactive molecules, too, are essential for life, but in excess are denoted as “oxidative stress” and often associated with many diseases. The few studies that have examined redox biology parameters have often utilized outdated and nonspecific methods, as well as have reported confounding results, which has made it difficult to conclude the role for redox in PTSD. Herein, we provide a foundation of how redox biology may underlie diseases like PTSD, critically examine redox studies of PTSD, and provide future directions the field can implement to enhance standardization, reproducibility, and accuracy of redox assessments for the use of diagnosis, prognosis, and therapy of this debilitating mental health disorder.

## Introduction

“I didn't feel empty. I wished I'd felt empty. ... I wanted to be empty like an overturned pitcher. But I was full like a stone.”

― Jonathan Safran Foer, Extremely Loud & Incredibly Close

The excerpt above, from fictional novel Extremely Loud and Incredibly Close, delves into the mind of a young boy, recently bereaved of his father lost in the Twin Towers, and his grandfather, a World War II veteran. Both characters exhibited depressive spirals, anxiety attacks, frequent flashbacks to the events, and reclusiveness from society, all classic symptoms of post-traumatic stress disorder. Post-traumatic stress disorder, commonly abbreviated PTSD, is a psychiatric disorder that manifests after experiencing or witnessing a traumatic event, such as combat exposure, sexual assault, or serious incident, such as a terrorist attack, or gun violence. While the term “PTSD” may be relatively contemporary, PTSD has long been described since antiquity. PTSD-like symptoms have been recounted in many ancient epics, such as the Epic of Gilgamesh and the Bible, and labeled under many aliases throughout historical literature (Crocq and Crocq, 2000). PTSD is estimated to affect over 6% of the American population, but even with this high prevalence, diagnostic criteria for this disorder are highly diverse and heterogenous (Kessler et al., 2005). In fact, there are over 636,000 ways to be diagnosed with PTSD using the current standard guidelines, and the field still lacks any definitive biological signatures to accurately diagnose or categorize the disease (Galatzer-Levy and Bryant, 2013). This clearly outlines the need for more advanced, objective, and decisive diagnostic criteria for such a debilitating mental health disorder.

Mental health disorders are often difficult to diagnose due to overlapping symptomologies and heterogeneous manifestations in patients. Many psychopathologies are often diagnosed by clinical interpretation of symptoms based on patient histories, which can be wrought with subjectivity. Patient questionnaires and screeners offer an additional diagnostic tool for mental healthcare providers, but again rely on patient interpretation and reporting—which may be misconstrued—leading to inaccurate diagnoses and treatment options. As alluded to, PTSD suffers from these difficulties in diagnosis as well, and remains diagnosed primarily *via* clinical assessment and guidelines set by the Diagnostic and Statistical Manual of Mental Disorders 5 (DSM-5). However, with the advent of large “omics” screens, promising molecular signatures have been identified that may help biologically profile this complex psychological disease. One recurring theme among many of these molecular signatures in PTSD is a link to redox biology, but controversy abounds in the literature regarding the reliability and reproducibility of these potential diagnostic markers. Furthermore, the complex and potential mechanistic role in which redox biology may participate in PTSD remains to be explored. This review will delve into the implications of redox biology in PTSD, discuss and critique the redox markers examined in PTSD to date, as well as suggest the appropriate measures necessary to accurately examine redox species as markers of this debilitating disease.

## Redox biology 101

Before investigating the possible crucial role of redox biology in mental health disorders and their potential diagnostic role in PTSD, one must appreciate the depth of redox biology itself, starting with the basics of free radicals and reactive species.

Organic free radicals, or organic species containing at least one unpaired electron, were discovered over 100 years ago by chemist Moses Gomberg (Henderson, 2000). While inorganic free radical species were well-accepted to exist before Gomberg’s discovery, the concept of biological free radicals was only theoretical and believed to be unlikely given the reactive nature of these species. In contrast, since their discovery, free radicals of many origins have been found to participate in biochemical reactions across all taxa. Research has uncovered links between free radical species in many diseases such as obesity, autoimmune diseases, and cancer (see reviews (Valko et al., 2007; Pham-Huy et al., 2008; Gill et al., 2016)), thus, these species were considered only unfavorable in biological contexts for many decades. However, it is becoming increasingly evident that free radical species are not all detrimental, but essential for countless redox reactions that are at the epicenter of life itself.

### What is redox?

For the non-expert, the field of redox biology and its terminology can be difficult to interpret. To begin, the term redox is a simple portmanteau of the words “reduction” and “oxidation.” Reduction is the act of giving an electron to another species, whereas a species is reduced when they have received an electron. Conversely, oxidation is the act of taking an electron from another species, whereas a species is oxidized when they have lost an electron. Given this base definition, redox reactions involve virtually every known biological reaction. The simple process of making a covalent bond between two species requires the transfer of electrons, which is the core of redox biology. However, redox biology rarely is associated with simply any biological process that requires the transfer of electrons, and is more often considered the field of biology associated with the study of reactive oxygen species (ROS) and free radicals.

As previously discussed, the term “free radical” refers to any molecule that has at least one unpaired electron (Lobo et al., 2010). Since electrons thermodynamically prefer to have a paired electron in each orbital, free radicals are often highly reactive and seek to scavenge or offload an electron to any nearby source. In the worst cases, this nearby source may be a critical cellular component such as DNA, protein, or lipids that could be damaged by electron transfer. In the best cases, this nearby source may be a species designed to counteract the reactivity of free radical species known as an “antioxidant.” These aptly named species react with free radicals to result in less reactive or non-reactive species, thus minimizing the potential for catastrophic cellular damage (i.e., oxidative stress).

There are several subcategories of reactive species, each corresponding to the central element involved in the redox reaction. The most common of these are ROS, which are oxygen-containing molecules that can participate in electron transfer reactions. Other categories of reactive species include reactive nitrogen (RNS), reactive thiyl (RSS), and reactive carbon (RCS) species, which at their core contain reactive nitrogen, sulfur, and carbon, respectively. It should be noted that not all reactive species are necessarily free radicals, with some existing in a highly reactive state even while possessing balanced electron orbitals. It is this complexity and variable levels of stability that make for a highly intricate system of both apparently random and tightly regulated molecular reactions.

### Types of reactive species and their formation

The broad family of ROS includes important species such as superoxide (O_2_^•-^), hydrogen peroxide (H_2_O_2_), and hydroxyl radical (^•^OH), among others. O_2_^•-^ is often the first of the biological ROS to be formed since it only requires a one electron addition to molecular oxygen. The formation of O_2_^•-^ may occur inadvertently due to electron leak from complexes of the mitochondrial electron transport chain, as a secondary product of the enzyme xanthine oxidase, or even as the primary product of the NADPH oxidase family of enzymes (Wong et al., 2017; Numan and Jamil, 2022). Because of the extra unpaired electron, O_2_^•-^ is highly reactive, which leads to damaging reactions with cellular components to create new organic reactive compounds or even other ROS, such as H_2_O_2_.

H_2_O_2_ is considered a ROS, but contains fully paired electron orbitals. Thus, H_2_O_2_ is not a free radical, less reactive than O_2_^•-^, and quite stable (even able to be bottled and purchased). While H_2_O_2_ can be formed by certain enzymatic processes such as xanthine oxidase, NADPH oxidase 4, and monoamine oxidases (Simonson et al., 1993; Kelley et al., 2010; Nisimoto et al., 2014), it is more often formed secondarily from O_2_^•-^. The conversion of O_2_^•-^ into H_2_O_2_ may occur by spontaneous dismutation or at a much faster rate by enzymatic dismutation using a class of enzymes known as superoxide dismutases (Case, 2017). Because of the stability of H_2_O_2_, it is maintained in cells at approximately 1–100 nM and is now considered a vital cellular signaling molecule (Sies, 2017; Sies and Jones, 2020). However, the reaction of H_2_O_2_ with free iron, also known as the Fenton reaction, creates the incredibly reactive and damaging ^•^OH. This free radical ROS is one of the most highly reactive biological radicals, and will react at a diffusion limited rate with virtually any biological substance causing widespread cellular damage. To date, there are no known enzymatic processes that purposefully generate ^•^OH, and its creation by the Fenton reaction is likely one reason why free iron is often highly sequestered inside cells.

Another class of reactive species are the RNS, which some examples include nitric oxide (^•^NO) and peroxynitrite (ONOO^−^). ^•^NO is by far the most predominant and well-studied RNS, and is deliberately produced through nitric oxide synthase enzymes. While ^•^NO is known for its ability to cause vasodilation in the cardiovascular system, it also serves as a very prominent and critical redox signaling molecule in virtually every cell type. Interestingly, ^•^NO is a relatively stable free radical and able to diffuse past membranes with minimal reactivity. However, ^•^NO and O_2_^•-^ react together at an incredibly fast, diffusion-limited reaction to form the reactive species ONOO^−^. ONOO^−^ is a highly oxidizing molecule known to react with and nitrate various biological species, primarily tyrosine moieties on proteins. Given that there are no known enzymes that directly generate ONOO^−^ or any systems to reverse the effects of tyrosine nitration, it is highly unlikely that ONOO^−^ serves any functional biological or signaling role like ^•^NO (Pacher et al., 2007; Ahmad et al., 2019).

The last two categories of reactive species to discuss are RSS and RCS. Both RSS and RCS are typically made as a consequence of ROS or RNS reacting with amino acids on proteins or carbons in polyunsaturated fats or sugars (Semchyshyn, 2014), thus they are not primary reactive species purposefully generated by cells. The formation of RSS typically leads to protein degradation, whereas RCS can lead to significant cellular damage due to the propagation of radical formation with nearby carbon moieties. Due to the wide array of thiol and carbon species in a cell, there are countless RSS and RCS species that may be formed in the presence of abundant ROS or RNS. Therefore, with all of these reactive species present within cells, one may wonder, how do biological organisms survive given these highly oxidizing conditions that steal electrons from critical cellular components? The answer to this quandary is simple: antioxidants.

### Redox balance: Antioxidants

To cope with the influx of oxygen into the atmosphere as a result of photosynthesis, primitive life utilized small molecules and enzymes to reduce reactive species into less damaging species (i.e., antioxidants), which we and others have comprehensively reviewed elsewhere (Matés, 2000; Kurutas, 2016; Case, 2017). Evolutionarily speaking, one of the first reported group of enzymes to have such antioxidant activity are metal-containing enzymes called superoxide dismutases, which catalyze the reduction of O_2_^•-^ into H_2_O_2_ and molecular oxygen. As previously mentioned, H_2_O_2_ may also inflict cellular damage, and thus several enzymes such as catalase, the paraoxonases (Pon), the peroxiredoxins (Prx), and the glutathione peroxidases (Gpx) have evolved to reduce peroxides into inert products (Lubos et al., 2011; Taler-Verčič et al., 2020). These enzymes may be found both inside and outside of the cell, as well as possess specific subcellular localization. This intricate and complex system of antioxidant enzymes demonstrates the importance of these reducing systems to maintain a constant electron rich environment and prevent oxidation.

In addition to enzymes, cellular systems also rely on electron rich small molecules as antioxidants. For example, α-tocopherol and ascorbate (vitamin E and C, respectively), exogenous antioxidants, aid in the reduction of lipid peroxides. This type of antioxidant intervention is critical, as it prevents the feed forward radical propagation reaction of lipid peroxides that would ultimately end in cellular death due to membrane oxidation (Fruhwirth and Hermetter, 2008; Traber and Stevens, 2011). Additionally, another group of antioxidants called endogenous antioxidants are non-enzymatic molecules that are naturally produced by the body. Endogenous antioxidants include uric acid, albumin, glutathione, bilirubin, among others. Uric acid is a byproduct of purine metabolism, and is known for scavenging ONOO^−^ in conjunction with vitamin C (Nimse and Pal, 2015; El Ridi and Tallima, 2017). Circulating albumin may also possess antioxidant properties. Due to its structure, albumin can sequester transition metals, carry bilirubin, and trap reactive species such as H_2_O_2_, ONOO^−^, and hypochlorous acid (Taverna et al., 2013). Bilirubin is made as a secondary breakdown product of heme catabolism, and can prevent lipid peroxidation (Stocker et al., 1987). Last, glutathione (GSH) is an antioxidant consisting of three amino acids: glutamine, cysteine, and glycine. GSH serves as a co-factor for antioxidant enzymes including glutathione peroxidase, which functions to detoxify peroxides, as well as glutathione-s-transferase, where it functions to reverse oxidized protein residues (Aquilano et al., 2014). The ubiquitous nature of these endogenous antioxidants indicates their necessity for generalized cell detoxification. A summary of reactive species and their respective antioxidants may be seen in Table 1.

**TABLE 1:** Antioxidant enzymes and redox targets.

Antioxidant	Method of action	Redox Target	Cellular Location	Notes
Peroxiredoxin (Prx1-6)	Enzymatic	H_2_O_2,_ hydroperoxides, ONOO^−^	Mt and cytosol	Works in conjunction with thioredoxin
Catalase (CAT)	Enzymatic	H_2_O_2_	Peroxisome	
Glutathione peroxidase (GPx1-7)	Enzymatic	H_2_O_2_ and hydroperoxides	Mt, nuclei and cytosol	Works in conjunction with glutathione
Superoxide dismutase 1 (SOD1)	Enzymatic	O_2_^•-^	Cytosol	
Superoxide dismutase 2 (SOD2)	Enzymatic	O_2_^•-^	Mt	
Superoxide dismutase 3 (SOD3)	Enzymatic	O_2_^•-^	Extracellular	
α-tocopherol (Vitamin E)	Non-enzymatic	Lipid radicals	Cell membranes	
Ascorbic acid (Vitamin C)	Non-enzymatic	Oxidized vitamin E	Cytosol	
Glutathione transferase (GST)	Enzymatic	Oxidized sulfur	Cytosol	Works in conjunction with glutathione

Summarizes several antioxidants and their respective oxidative species they target, as well as cellular location and required secondary co-factors (if any). Mt: mitochondria.

### Redox fossils as biomarkers

There is no denying that free radicals and reactive species have the potential to be harmful to cells and organisms, however, it is now highly accepted that these molecules also play critical signaling roles in normal physiology (Valko et al., 2007; Schieber and Chandel, 2014). One primary characteristic of any signaling molecule is the ability for its modification to be reversible, so as to restore homeostasis after the signal has dissipated. This is why reactive species signaling must be so tightly regulated. Too few reactive species lead to not enough amplitude to create a signal, but too many reactive species lead to irreversible oxidative damage that reaches beyond the realm of normal reversible signaling (i.e., oxidative stress). It is the latter of these two that often occurs in disease, and oxidative damage has been reported in virtually every disease known to man. This irreversible oxidative damage often leaves behind unique molecule(s) or signatures, and these “fossils” are often molecules of interest used as biomarkers of oxidative stress in diseases.

#### Lipids

The most commonly examined redox biomarkers are products of lipid peroxidation. Lipid peroxidation occurs when an oxidant attacks a carbon double bond in a lipid, forms a lipid radical that reacts with oxygen, and produces a lipid peroxide. Normally, these lipid peroxides are terminated by an antioxidant intermediary, but in disease states these peroxides may perpetuate a feed-forward chain-like reaction of lipid radical formation. Once in an oxidized state, these modified lipids may form secondary deleterious products such malondialdehyde (MDA), 4-hydroxynonenal (4-HNE), and F_2_-isoprostanes, which may lead to the destruction of cell membranes and cell death (Ayala et al., 2014).

MDA is commonly used as a biomarker of lipid peroxidation given the ease of measurement. There are numerous commercial MDA enzyme-linked immunosorbent assay (ELISA) kits on the market that claim specific antibodies towards MDA. Additionally, MDA may be measured using a thiobarbituric acid reactive substances (TBARS) assay where MDA reacts directly with a colorimetric substance that may be detected spectrophotometrically. However, it must be noted that both methods are often not specific in solely reporting MDA, and often can inaccurately detect other substances such as other aldehydes, proteins, alkanals, and urea present in the samples (Steppeler et al., 2016; Murphy et al., 2022). Furthermore, even if MDA is detected using more advanced technology such as mass spectrometry, it must be considered that MDA is not solely generated from oxidative stress or disease. MDA has been shown to be formed *via* other normal cellular reactions with metal ions, as well as a side-product metabolite from prostaglandin synthesis (Smith et al., 1976; Poznyak et al., 2020; Wang et al., 2021). Moreover, MDA levels in healthy patients have also been known to be extremely variable, thus making it difficult to discern any significant differences between healthy individuals and those with disease (Khoubnasabjafari et al., 2015).

Another secondary lipid peroxide product is 4-HNE, which results from the decomposition of unstable n-6 lipid radicals (Csala et al., 2015). This toxic reactive molecule, like other reactive species, can interact with DNA, other lipids, and proteins, and is even known to inhibit DNA repair mechanisms (Feng et al., 2004). Similar to MDA, 4-HNE is often measured indirectly *via* ELISA, in which HNE-conjugated proteins are captured and colorimetrically measured. 4-HNE is metabolically processed quickly, with a physiological half-life of under 2 min (Dalleau et al., 2013), thus methods of detection that minimize handling time (such as HPLC or GC-MS) should be used to measure this compound more accurately. However, these methods are not often available in clinical settings, which limits the utility of this biomarker (Dham et al., 2021). Additionally, 4-HNE can be absorbed directly through dietary sources, thus careful attention to patient’s diet should be noted when comparing individuals (Guillén and Goicoechea, 2008; Csallany et al., 2015; Steppeler et al., 2016).

The last lipid peroxidation product that is often measured to represent oxidative stress are F_2_-isoprostanes, which are secondary products from the oxidation of arachidonic acid (Morrow et al., 1990). These molecules are known to induce vasoconstriction as well as inflammation (Kaviarasan et al., 2009). F_2_-isoprostanes are most accurately measured using LC/MS or HPLC, but these assays are costly and low throughput. Other assays including radioimmunoassays and ELISAs have been developed, but possess similar issues in specificity as previously discussed. Like the other secondary lipid peroxidation products, F_2_-isoprostanes are degraded quickly, with a half-life of 4 min in plasma samples (Kaviarasan et al., 2009). Also similarly, F_2_-isoprostanes are known to be elevated in certain biological settings and environmental sources. For instance, one studied showed that pregnant women had significantly higher levels of F_2_-isoprostanes in the blood compared to non-pregnant women (Morris et al., 1998), while another studied showed a significant increase of F_2_-isoprostanes in the blood of aged rats compared to young rats, indicating the importance of appropriate control populations (Roberts and Reckelhoff, 2001).

In conclusion, while using secondary lipid oxidation markers as biomarkers is simplistic, it comes with many caveats, as these molecules often form from various sources, degrade quickly in serum, and methods to measure are often expensive or unspecific. Therefore, extreme caution should be exercised by the reader when secondary lipid peroxidation markers are used solely as a read out of oxidative stress. Furthermore, researchers should consider secondary methods of validation for lipid peroxidation in addition to this primary screening method.

#### Proteins

In addition to oxidation of lipids, reactive species often oxidize proteins, leading to carbonyl group (ketones, aldehydes) formation on amino acid side chains. These carbonyl groups may cause the protein to unfold, leading to a nonfunctional conformation (Reeg and Grune, 2015). Like lipid peroxidation, measuring protein carbonyl groups also poses issues regarding specificity and determining the cause of the carbonyl group formation. While carbonyl groups on proteins may be formed *via* proteins reacting with reactive species, they are also known to form as a consequence of protein glycation, or the reaction of sugars with proteins, also known as the Maillard reaction (Dalle-Donne et al., 2003). These stable reactive carbonyl groups can be directly measured using 2,4-dinitrophenylhydrazin, ELISA, or other antibody-based methods, however one should note that not all oxidized proteins contain carbonyl moieties, and thus will not be measured with these methods (Murphy et al., 2022). Other factors should be considered when utilizing protein carbonyls as a measure of oxidative stress for certain diseases. For instance, patients with Diabetes Mellitus, which is known to affect 10% of Americans (CDC, 2022), have significantly upregulated levels of protein carbonyl groups in their blood compared to healthy controls likely due to the Maillard reaction as opposed to reactive species stress (Almogbel and Rasheed, 2017). Thus, additional consideration should be given to pre-existing health conditions of the patient when utilizing protein carbonyl levels as an assessment of oxidative damage.

Another element on proteins that is susceptible to oxidative modification is sulfur. Reactive species may react with sulfur containing amino acids, which can result in structural, spatial, or functional changes on proteins. Unlike protein carbonyls, sulfur modifications on proteins are known signaling modifications and equate to a vital means of protein homeostasis (Paulsen and Carroll, 2013). These oxidized sulfurs can be reversed to their reduced form by the enzyme glutathione S-transferase with co-factor glutathione, or by the specialized sulfenic reducing enzyme, sulfiredoxin. In contrast, excessive amounts of reactive species (i.e., oxidative stress) can lead to multiple oxidation events, creating irreversible protein alterations and species such as sulfonic acid and sulfonamide, which can ultimately target proteins for degradation by proteosomes (Kehm et al., 2021). Measuring these species is not trivial, and often requires advanced techniques involving spin-trapping and mass spectrometry (Hawkins and Davies, 2019). However, deciphering the exact oxidative modification on protein sulfur residues is exactly what is required to understand physiological redox signaling versus oxidative stress, and is the direction the field needs to move to gain a greater understanding of how redox shapes different pathological states.

As mentioned above, ONOO^−^ formation is the result of O_2_^•-^ reacting with ^•^NO. ONOO^−^, which has no known direct biological function *in vivo*, may in turn nitrate tyrosines on proteins. Protein nitration is a strong, covalent modification which can affect protein function and structure (Cipak Gasparovic et al., 2017). As such, these nitrated products can be measured to reflect nitrosative stress in cells. Specific antibodies against nitrotyrosine can be used in Western blot assays, immunohistochemistry, and ELISAs to measure nitrosylated proteins, although commercial antibodies have varying sensitivities in detection (Petre et al., 2008). Moreover, many of these assays simply measure total protein nitration, which does not lend insight into specific protein modifications or sources of ONOO^−^. ONOO^−^ itself can be measured using fluorescent probes, such as dihydroxyrhodamine, but this probe is known to also react with other radicals, which limits its specificity for ONOO^−^ (Ríos et al., 2017). Newer probes that rely on boron at the active site do react with ONOO^−^ at a very fast rate (Chen et al., 2013; Sedgwick et al., 2016), however, the high electrophilic reactivity of ONOO^−^ makes it difficult to find competitive detection methods that are specific for this one reactive species.

#### Nucleic acids

The last major biological signature of oxidative stress is oxidative damage of DNA. Some DNA bases, such as guanine, can be oxidized by reactive oxygen species leading to the formation of 8-hydroxydeoxyguanosine (8-oxo-dG). This oxidation of the guanine base is typically excised and repaired quickly by specialized base excision repair enzymes. However, if the mutation is not repaired, transversion or transition mutations in the DNA can occur resulting in a permanent mutation in the DNA (Poetsch, 2020). Furthermore, oxidation to the free guanine pool may also occur leading to damaged nucleotides even prior to incorporation into DNA (Sekiguchi and Tsuzuki, 2002). 8-oxo-dG may be measured by a variety of assays including high performance liquid chromatography (HPLC), mass spectrometry, or by various antibody detection methods. One limitation of these assays is that the half-life of 8-oxo-dG is approximately 11 min, thus detecting significant quantities may prove difficult dependent upon the experimental system (Hamilton et al., 2001). Additionally, DNA may also be easily oxidized in sample preparation steps, thus it is necessary to have proper controls to compare baseline 8-oxo-dG levels and avoid experimental artifacts (Hamilton et al., 2001).

In addition to oxidized DNA bases, reactive species may also cause double and single stranded DNA breaks, affect chromatin remodeling complexes, and alter epigenetic modifications (Srinivas et al., 2019). All of these types of DNA damage or modification may be measured using standard molecular biology techniques, but again, caution must be taken regarding the origin of the damage. All of these modifications to DNA may be caused by factors other than reactive species, therefore, concluding these damages are a result of oxidative stress must be supported with additional evidence of redox etiologies.

#### Antioxidants

While oxidant fossils potentially give insight into oxidative damage of cellular components, the assessment of antioxidants also may provide an understanding to the cause or progression of a pathology. Antioxidants can be measured at both a concentration level, and a capacity level, or the efficiency in which reactive species are converted by antioxidants (Apak et al., 2016). The concentration of antioxidants is often assessed using antibody specific assays, while antioxidant capacity can be measured using hydrogen atom transfer reaction assays or single electron transfer reaction assays (Huang et al., 2005). Brief examples of commonly assessed antioxidants and assays to quantify them are discussed below, while more comprehensive reviews are listed here (Huang et al., 2005; Alam et al., 2013; Xiao et al., 2020).

Given the extensive antioxidant role of glutathione in cells, it is often quantified as a measure of redox status of cells. In the processes of detoxifying cells, reduced glutathione (GSH) donates its electrons to an oxidant, but becomes oxidized in the process to oxidized glutathione (GSSG). Thus, many assays that quantify the ratio of GSH/GSSG have been developed, including assays that measure GSSG by preventing the oxidation/reduction cycle (using agents such as N-ethylmaleimide (NEM) and vinylpyridine) so as to understand the relative concentration of both forms at any time in a given system (see (Zitka et al., 2012) for an extensive review on measuring glutathione). One major limitation with this method is that significant oxidation is needed to occur to greatly disrupt the GSH/GSSG balance of a cell. Therefore, small perturbations in redox changes that may be relevant biologically may not be detected by this assay alone.

In addition to measuring antioxidant cofactors, the capacity and concentration of antioxidant enzymes, such as glutathione peroxidases, peroxiredoxins, catalase, and superoxide dismutases can reflect the redox environment of cells as well. Concentrations of these enzymes are often measured using ELISA and Western blot analysis. However, concentration alone of these antioxidant proteins means little to their overall ability to detoxify reactive species. Many posttranslational modifications have been shown to alter antioxidant enzyme activity, thus assessment of only the protein quantity limits the ability to draw appropriate conclusions. Therefore, it is critical that any type of antioxidant enzyme analysis contains both protein concentration and enzyme activity. Together, these allow for specific activity of these enzymes to be determined, which allows for a broader understanding a particular antioxidant environment.

Another common and simple method used in the literature to assess antioxidants are kits/assays that measure total antioxidant capacity of a specific system. These types of assays often rely on the color change of a reactive species such as 3,3′,5,5′-tetramethylbenzidine (TMB), which can be oxidized and reduced respectively by pro-oxidants and antioxidants to reveal a ratio of redox status (Alamdari et al., 2008). This method may be used to determine the crude oxidant versus antioxidant cellular environment, but fails to shed light on possible causative enzymes or molecules that may be responsible for this phenotype.

In addition to what was mentioned above, all of these assays are susceptible to unwanted artifacts given the method of sample preparation. Investigators should be highly mindful of the biological sample, source, or pre-existing condition prior to the assessment of any type of antioxidant, and the appropriate steps should be taken to mitigate unwanted or artificial results (Ghiselli et al., 2000). In conclusion, measuring redox fossils in a standardized fashion is an appropriate first step in garnering a broad and unfocused image regarding the redox environment. However, because these fossils are indeed relics of the past, they cannot provide the intricate details of how redox species are shaping physiology in a specific system. Given these complications and limitations, their validity must be questioned.

#### Are redox biomarkers viable?

Biomarkers are described as any molecule or substance that can be objectively and repeatedly measured to predict a specific disease (Strimbu and Tavel, 2010). The best biomarkers are those that consistently correlate with a distinct disease and are easily measurable in a patient’s bodily fluids. This makes the utility of redox biomarkers often problematic, as their origin may be highly localized inside cells or tissues as opposed to being present systemically at detectable levels. As previously discussed, many stable redox fossils that are easily measured often do not provide enough information regarding the specific changes being made to the redox environment. Additionally, many diseases are known to have oxidative stress components, thus limiting the ability of redox biomarkers to differentiate particular disease states. However, while redox biomarkers may not be viable alone, in combination with other diagnostic criteria, more specific redox biomarkers may be able to predict severity, progression, or subtypes of disease that are currently uncharacterized. Furthermore, the assessment of redox should not only be the fossils, but attempts should also be made to directly measure the redox species themselves to further understand the redox system within a specific disease. This may be very important in complex diseases where multiple risk factors contribute to the development of the condition, and one prime example of this may be post-traumatic stress disorder (PTSD).

## Redox and non-redox biomarkers of PTSD

To date, the potential biomarkers identified for PTSD primarily focus on the brain. However, given the inability to physically and safely access the brain in living patients, many of these brain biomarkers are invasive (e.g., cerebrospinal fluid samples) and expensive to measure (e.g., functional magnetic resonance imaging, MRI). Due to this, the field has exclusively relied on more subjective measures of diagnosis including clinical interpretation or questionnaires. Thus, the quest for non-brain derived biomarkers for PTSD diagnosis has been a high priority goal of the field.

Specifically regarding redox, one early study attempted to examine redox parameters as potential predictors of severity of PTSD as based by the Clinician-Administered PTSD Scale (CAPS), which is a 30-item structured interview linked to the DSM-5 criteria for PTSD (Tezcan et al., 2003). In a cohort of 14 PTSD patients and 14 healthy controls, the researchers examined antioxidant function and MDA levels in erythrocytes. While the study found no differences in these factors between PTSD and healthy patients, they did observe that GPx and SOD activities were significantly and positively correlated with CAPS scores (MDA and catalase activities were not). Interestingly, the authors concluded from these results that the production of free radicals do not seem to be related to PTSD, however we disagree, and believe this cannot be concluded given their dataset. First, while this study does control for certain factors including age, sex, medications, and other mental health diseases, the small sample size, the vast range of PTSD duration, CAPS scores, and only examining red blood cells limits the study’s findings and ability to cast such a broad conclusion. Furthermore, the authors did not directly measure free radicals, but only antioxidant enzyme activities and MDA, which aforementioned is a problematic downstream product of lipid peroxidation and other lipid processes. Last, the positive correlation between GPx/SOD activities with CAPS scores does in fact suggest the possibility of redox moieties in PTSD, but additional studies would be needed to further decipher this link.

In another study, redox markers including 8-oxo-dG, serum thromboxane B2 (a metabolite of arachidonic acid), protein carbonyls, total protein, albumin, and urate levels were analyzed in urine or serum samples between 46 military males with PTSD and 28 healthy military male controls (Ceprnja et al., 2011). While the study initially concluded there was a significant decrease in both protein carbonyls as well as albumin levels in the serum of PTSD patients compared to controls, these two markers failed to distinguish the two groups using a predictive receiver operator curve (ROC) analysis. It should be noted that all PTSD patients in this study were currently taking selective serotonin reuptake inhibitors (SSRIs) to control PTSD symptoms, which has been shown to reduce protein carbonyls in stressed rats (Zafir et al., 2009). Additionally, most parameters measured in this study were solely measured by ELISA methods, which as mentioned before, are notoriously non-specific (Murphy et al., 2022). Lastly, it is known that PTSD severity and symptomology can vary with time post the initial traumatic event. The samples in this study were taken from the patients 15 years after their service had ended, hence interpretations of this data cannot accurately be compared to PTSD patients with recent trauma exposure.

In effort to identify a blood-based biomarker for PTSD, Tylee et al. performed a pre- and three times post-deployment blood analysis of 50 male marines; half of which were clinically diagnosed with PTSD after returning home (Tylee et al., 2015). These types of studies are highly informative given the ability to utilize each individual as their own control before and after the development of PTSD. Using RNA isolated from whole peripheral blood mononuclear cells (PBMC) on gene array chips, the group identified that the top two gene transcripts that were most significantly downregulated in PTSD marines were GSTM1 and GSTM2. These two genes encode cytoplasmic mammalian glutathione S-transferases, which utilize glutathione to help detoxify the cellular environment. The expression of these genes is known to be altered under differential redox conditions to help combat excessive increases in reactive species (Nebert and Vasiliou, 2004), suggesting that, while not directly measured in this study, homeostatic levels of reactive species are likely perturbed in PTSD. The study went on to develop a support vector machine (SMV) model utilizing these two transcripts which predicted PTSD with an astounding 80% accuracy. This raises the question, with such a powerful potential biomarker, why are clinicians not using this biomarker today? Unfortunately, as mentioned in the discussion section of the study, other groups have found opposing results, conversely showing an increase in GSTM1 in PTSD patients (Neylan et al., 2011), making it difficult to interpret. Additionally, due to the small sample size and possible uncontrolled confounding factors, such as medication and pre-existing medical conditions, this potentially robust diagnostic marker is still not used today.

In a recent metadata analysis analyzing 54 human studies for bloodborne inflammatory and redox biomarkers of PTSD, only C-reactive protein, interleukin 6, and tumor necrosis factor reached statistical significance in patients with PTSD compared with healthy controls (Peruzzolo et al., 2022). Because of these results, the authors concluded that no oxidative stress marker associated with PTSD. However, of the 20 biomarkers analyzed, only three (MDA, paraoxonase 1, and catalase) were examined as potential redox markers. The measurement of only these three are problematic for several reasons. First, of the 54 studies that were included in the meta-analysis, only 2-3 studies measured these redox markers, severely decreasing the sample size and inherently decreasing the chance of reaching statistical significance. Second, the methodology measuring these respective oxidative stress markers differed between studies. Third, the biomarkers analyzed are spatially specific enzymes (without activity being measured, only protein level) and a problematic marker of lipid peroxidation, thus limiting the interpretation of these results. Last, differences in the time since patients experienced psychological trauma were observed across each of the individual studies. Taken together, while the findings of significant inflammatory proteins in PTSD is in fact interesting, these limitations should not discount the potential of redox mechanisms also possibly contributing to the disease.

Finally, a comprehensive discovery study was recently undertaken that examined over 1 million potential markers of PTSD including proteins, DNA methylation, single nucleotide polymorphisms, miRNAs, redox markers, immune cell populations, cytokines, and metabolomic data (Dean et al., 2020). From this massive undertaking, the authors homed in on 28 biomarkers that performed with 81% predictive accuracy in a separate validation cohort. In the study, five indirect redox markers were measured: F_2_ isoprostanes, 8-oxo-dG, glutathione (GSH/GSSH), Gpx, and vitamin C. None of these markers were retained in the final 28 predictive biomarker group. However, similar to previous studies, these markers focus on general redox fossils of oxidized lipids and nucleotides (F_2_ Isoprostanes, 8-OH-dG) and peroxide antioxidants (GSH and glutathione peroxidase), and thus do not capture the entire redox picture that is occurring in PTSD patients. The review concludes that redox changes as a whole are not reliable biomarkers for identifying patients with PTSD, but again, this view is flawed given the minimal examination performed regarding the breadth of redox environment and its regulation.

Overall, while many human studies have shown promise in developing a robust panel of biomarkers for diagnosing PTSD, they often fail to recognize the possible confounding factors of the actual markers assessed. Furthermore, the dimension of time since the trauma exposure is often rarely discussed or taken into analysis. Because of this, many studies conclude conflicting reports regarding the role of redox signaling in PTSD, which consequently convolutes the literature and disregards a possibly powerful diagnostic tool. To understand the dynamic interplay of redox, time, and space in this complex, multifaceted disease, better mechanistic models are needed to identify and track biomarkers that can ultimately alleviate, prevent, or aid the diagnosis of this debilitating disease.

## Further directions: What’s missing and how should we proceed?

Due to the variation in biological subjects, diverse manifestations, severity, and timeline of PTSD, identifying the possible role of redox markers as mechanistic regulators and/or biomarkers of PTSD poses challenges, but is not impossible. In order to confidently and consistently assess certain redox parameters, careful attention must be made to the timing and methodology of measurements, specific location and type of reactive species, and other demographical characteristics of the patient relevant to their trauma and PTSD. By establishing a homogenous protocol for measuring redox markers in patients, certain redox markers may prove to be powerful and definitive biomarkers that could allow for early therapeutic intervention to mitigate some of the physiological and psychological manifestations associated with PTSD.

### Time from the traumatic event

One unique aspect of PTSD is the ability to definitively determine the traumatic etiological event. If biomarker panels were developed that demonstrated how redox or non-redox biological markers changed over time after a traumatic event, this information could be utilized to understand the disease progression and treatment options. This type of methodology has already been applied to another disease with a known causal etiology, traumatic brain injury (TBI). For example, TBI patients have previously been shown to have elevated total ROS and lowered antioxidant capacity that is seen to peak around 5 days post-trauma, but lasts for only a week (Bjugstad et al., 2016). While the specific methods of measurement in this work pose similar problems to other non-specific redox measurements, the demonstration of temporal redox changes allows for the understanding of disease progression. Furthermore, TBI and PTSD often correlate, with one study indicating one-quarter of TBI patients were diagnosed with PTSD 6 months post-trauma (Bryant et al., 2000). While the study by Bjugstad (Bjugstad et al., 2016) and colleagues did not examine PTSD outcomes in with their TBI patients, future studies may identify that redox markers act as effective markers for the development of PTSD and could allow for earlier intervention.

The temporal and spatial components of PTSD have also been examined using animal models of PTSD. One study found significantly increased O_2_^•-^ and total ROS in blood samples and several brain regions in rats after predator exposure (Wilson et al., 2013). In another rodent model of PTSD, Duan and colleagues utilized mass spectrometry to identify proteins differentially expressed in the hippocampus of resilient versus susceptible rat populations within a few days after fear extinction test (Duan et al., 2021). Subsequent pathway analysis revealed susceptible populations had perturbed glutathione binding, oxidation-reduction processes, and even determined Gstm2 - the same gene implicated to be downregulated in PTSD patients in the study by Tylee et al. - to be differentially altered in the susceptible mice. Additionally, work from our lab using a preclinical mouse model of PTSD known as repeated social defeat stress (RSDS) demonstrated that mitochondrial O_2_^•-^ specifically in circulating and splenic T-lymphocytes is elevated immediately after psychological trauma and correlated with PTSD-like behaviors (Moshfegh et al., 2019). Subsequent single cell RNA sequencing data revealed that T-lymphocytes isolated from stressed animals had a significant upregulation in gene expression pathways involved in oxidative phosphorylation and mitochondrial dysfunction, further highlighting the possible redox connection in immune cells in PTSD animal models (Moshfegh et al., 2022). One possible reason these animal studies show strong redox signatures with psychological trauma compared to the human studies is the control of time after the traumatic incident. Many human PTSD studies fail to consider the variable of time since the traumatic incident when evaluating redox markers—a variable that is easily controlled in animal research. Thus, it is crucial that time since the traumatic event is considered and controlled for when measuring redox parameters as potential biomarkers for human PTSD.

### Patient background

As previously discussed, altered redox state is known to occur in a plethora of diseases, including obesity, cardiovascular diseases, neurological diseases, Type II diabetes, and metabolic syndromes (Korac et al., 2021). Over 400 million people in the world have Type II diabetes and roughly 650 million people are obese, thus controlling for underlying diseases and body makeup that may influence the results of redox assays is imperative for determining accurate redox biomarkers (Khan et al., 2020; World Health Organization, 2021). Additionally, some medications are also known to inherently perturb the redox environment. The current recommended medications for PTSD patients include SSRIs, which are seen to directly influence some measurable redox markers such as 8-oxo-DG (Ștefan et al., 2020; Jorgensen et al., 2022)*.* Blood pressure medications, such as statins and angiotensin II antagonists may also affect NADPH oxidases, which can alter O_2_^•-^ production from these enzymes (McCarty et al., 2015). Other medications may directly target antioxidant transcription factors or inhibit other pro-oxidases, ultimately skewing the results of studies that fail to control for these types of medications (Dao et al., 2015). Together, these observations demonstrate how important patient demographics are regarding the redox environment, and how a more nuanced approach is desperately needed to understand this field with greater appreciation.

Regarding mental health specifically, there have been extensive debates regarding how these types of diseases are currently classified and diagnosed. Similar to PTSD, there are thousands of different ways categorical mental health diseases such as depression, anxiety, and others may be currently diagnosed. This lack of specificity or refinement of diagnosis places individuals of significant varying degrees of disease into ambiguous large disease categories where treatment regimens are standardized and often fail. For this reason, the Research Domain Criteria (RDoC) initiative was formed by the National Institute of Mental Health, which aims at creating a more nuanced dimensional approach. With the RDoC framework, dimensions of behavior, environment, and physiology are used to assess individuals as opposed to binning using categorical diseases (Morris and Cuthbert, 2012). The ultimate goal of this research initiative is to further understand the complexity of mental health disorders, and to potentially identify possible subtypes or groups within the larger categories. Biomarkers may play a large role in this initiative, as these biological signatures may aid in the differentiation of two types of patients diagnosed with the similar disease (Goossens et al., 2015). This methodology has already been used extensively in the cancer field, where tumors are regularly characterized for specific tumor markers that aid in diagnosis and treatment. These types of analyses are very possible for redox biomarkers, and should be considered as a possible new dimension of analysis in studies moving forward.

### Methods of measurement

There are an abundance of methods for measuring reactive species, which may be both advantageous and detrimental. Biomarkers require definitive measurements that allow for high specificity and sensitivity. Many redox assays for biomarkers are packaged as easy-to-use kits, which enhances the availability and access to this technology, but often at a cost of non-specific and unreliable results (Murphy et al., 2022). Non-redox experts may also succumb to a lack of understanding of how to interpret what is being measured regarding the redox environment. For instance, if one study measures the gene expression of an antioxidant enzyme, and another study measures the enzymatic function of the same antioxidant protein, these two results could be completely different and yield opposite conclusions regarding the redox state of the disease assayed. Therefore, a firm understanding of what is being measured, the methodology utilized, and the interpretation of the results are paramount in the assessment of the redox environment in health and disease.

Most of the PTSD studies examined in this review utilize blood samples to measure redox parameters, which are often frozen for an unspecified amount of time before the assessment of various redox biomarkers. If not prepared correctly, freezing samples severely limits the validity of some reactive species assays. Moreover, the thawing process itself may cause the production of reactive species, which prevents the ability to compare fresh and frozen redox measurements (Polson et al., 2018; Pawlik-Sobecka et al., 2020). Additionally, the type of anticoagulant used for collecting patient blood samples, such as heparin, EDTA or sodium citrate can also affect the measurement of metabolites and antioxidants in samples taken from the same patient (Kennedy et al., 2021). Many studies also fail to assess a specific cell type when examining the blood. Plasma/serum are often used when looking for redox fossils, but because they contain no cells, provide no evidence as to the source of the original reactive species. Erythrocytes are also problematic, as they only have a half-life of approximately 120 days. Examining these cells may result in the loss of signal if the redox event is short lived, and the erythrocyte population has turned over. Last, many studies utilize PBMCs for analysis of redox parameters. While these cells are more appropriate given their ability to actively generate reactive species, they consist of multiple cell types that are often not examined individually. Bulk analysis of these cells causes a smaller signal to noise ratio similar to that observed with bulk RNA sequencing compared to single cell RNA sequencing. With all of these challenges, likely the most significant limitation in the previous studies is the lack of direct measurement of redox species. Direct measurement of redox species is the gold standard for understanding the redox environment, but it also comes with technical challenges. Because of this, it is likely why many in the field have defaulted to using easier, albeit nonspecific methods to assess redox parameters. Understanding the need for methods that are translational, scalable, as well as sensitive/specific, we propose two primary methods for directly measuring redox species and one specific method for precisely measuring redox fossils for the field of mental health moving forward.

The first method is known as electron paramagnetic resonance (EPR), which is the gold standard for free radical detection. EPR is a technology that relies on the magnetic properties of free radical species. The unpaired electron on these species makes them magnetic by definition, which EPR is able to detect. Furthermore, given the magnetic signature, EPR is highly specific in its ability to decipher different radical species (Davies, 2016). The use of spin traps and spin probes allows for the stabilization of free radical species, and these may also be targeted to specific organelles, cells, and tissues allowing for spatial resolution of free radical levels (Mason, 2016). EPR can be run on an array of solid or liquid substrates that are either fresh or frozen, which allows for its applicability to countless basic or clinical scenarios. Newer EPR machines have become much more user-friendly with cost-effective tabletop models also available, which has greatly expanded access to this technology. While EPR is undoubtedly one of the strongest tools in the redox toolbox, it also possesses limitations. First, EPR is only able to detect free radicals, therefore some reactive species are not able to be recognized. Workarounds using secondary molecules that convert some non-radical species into radicals have been developed, but not for all reactive species. Second, where EPR excels at specificity, it often lacks in sensitivity (Mason, 2016). Because of this, free radicals are often at levels below the limit of detection. The newer devices are often capable of enhancing these signals, but this still remains a significant issue with the technology. These limitations do not by any means outweigh the strengths of EPR, and it should be considered for any type of mental health redox biomarker study moving forward.

The second method we propose is the use of fluorescent redox-sensitive probes. The list of these types of probes is extensive, but range from probes that are oxidant-specific, organelle-specific, cell permeable/impermeable, color diversity, and much more (See Table 2). These types of probes may be used on an array of cell and tissue types, and can be analyzed by microscopy, flow cytometry, spectrophotometry, and other methodologies. Many of these probes directly react with reactive species, and are designed to be highly sensitive to one particular species over others. In contrast to EPR, fluorescent probes are abundantly sensitive and allow for the measurement of incredibly small amounts of reactive species (Jiang et al., 2018). However, that enhanced sensitivity comes at a cost of specificity, as many of these probes have been shown to react with an array of reactive species. Additionally, inappropriate titering of these probes may lead to inaccurate conclusions regarding subcellular localization. If the probe is given at too high/low concentrations, it may not reach its destined target and give false results (Wardman, 2007). Last, one limitation in the translation of these probes is that they need to be used on fresh cells or tissue, which often makes clinical work more difficult. However, this is by no means impossible, and these probes provide a highly user-friendly and translational method of directly detecting reactive species in a biological setting. In fact, our collaborators have recently utilized this exact technology to translate our animal work into humans with major depressive disorder. In this work, Grotle et al. utilize a fluorescent probe to specifically measure mitochondrial O_2_^•-^ in T-lymphocytes from these patients and demonstrate a significant increase in this reactive species in patients with depression compared to healthy controls (Grotle et al., 2022). While many studies are currently underway to further understand the role of this reactive species in these patients, this study is a pivotal first step in the next chapter of mental health redox assessment.

**TABLE 2:** Recommended fluorescent probes for assessing redox status.

Intended target	Probe Name	Location	Advantages	Disadvantages
O_2_^•-^	Dihydroethidium (DHE)	Cytoplasm	Quick reaction; bright fluorescence; sensitive	Non-specific oxidation product ethidium can intercalate into DNA, artificially increasing fluorescence
	NeoD		Does not intercalate into DNA	Not widely used
	MitoSOX Red	Mitochondria	Can be used in live cells; sensitive; bright fluorescence	Caution should be taken regarding probe concentration
	MitoNeoD		Does not intercalate into DNA	Not widely used
H_2_O_2_	HyPer7	Cytoplasm	Very sensitive to low concentrations of H_2_O_2_	Must be expressed endogenously as a transgene
	Peroxyorange (PO1)		Sensitive to low H_2_O_2_ concentrations	Can react with ONOO^−^, HOCl
	Amplex-Red	Extracellular	Easy to use	Signal can be bleached or artificially excited by surrounding light
				Other ROS can inactivate HRP
				Requires an enzyme to catalyze probe
	MitoPY1	Mitochondria	Spatial specificity	Caution should be taken regarding probe concentration
^•^NO	4-amino-5-methylamino-2′,7′-difluorofluorescein diacetate (DAF-FM)	Cytoplasm	Selective for NO, over ONOO^−^	Can be autoxidized in the presence of light (see ref (Balcerczyk et al. (2005)))
ONOO-	HKGreen	Cytoplasm	Highly sensitive for ONOO^−^ (see ref. (Peng et al. (2014)))	Weakly interacts with HOCl, OH

Summarizes different fluorescent redox probes that can be utilized to assess the redox status of aqueous solutions, live cells, or tissues. Detailed reviews focused on various redox probes can be found here (Wardman, 2007; Bai et al., 2019; Murphy et al., 2022).

**FIGURE 1:**
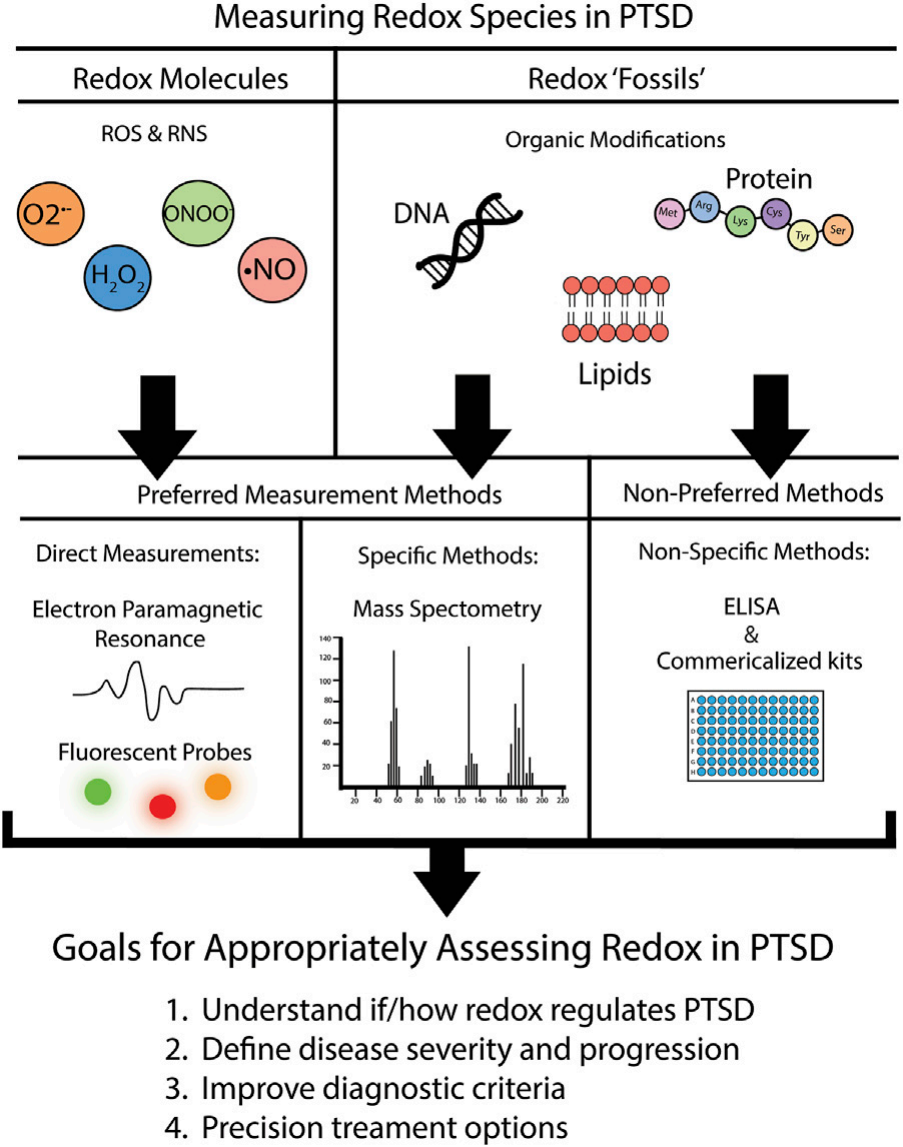
Recommendations for assessment of redox parameters in PTSD. We put forth recommendations of preferred versus non-preferred methods for the assessment of the redox environment. Further details on specific fluorescent probe recommendations are located in Table 2.

Last, measuring redox fossils can be a powerful addition to studying the role of redox biology in PTSD, but as stated throughout this review, is often quantified using non-specific methods, and thus incorrectly assessed. To increase the specificity of measurement, we propose using mass spectrometry (MS). Typically coupled with liquid or gas chromatography separation techniques, this method has been used to detect redox mediated protein modifications (Pan et al., 2014), antioxidant oxidation status (Lee et al., 2016; Burger et al., 2020), lipid oxidation products (see review (Hu et al., 2017)), as well as oxidized DNA byproducts (Chen et al., 2020). Mass spectrometry offers heighten sensitivity, and in one study measuring known MDA levels in prepared samples, MS showed up to a 6-fold increase in sensitivity using mass spectrometry over the typical TBARS assay (Liu et al., 1997). Thus, when feasible, mass spectrometry is the preferred method of measuring redox fossils due to the increased confidence compared to non-specific ELISAs and commercialized kits. Figure 1 summarizes our recommendations for researchers in the field regarding preferred redox assessment methods moving forward.

## Concluding remarks

The literature surrounding the quest for biomarkers of PTSD is far from complete, but with increasing ability to assess multi-omics parameters in large cohort samples, a promising biomarker list diagnosing PTSD may come to fruition soon. Pre-clinical research over the last decades has demonstrated an undeniable role of redox perturbations in the psychiatric disorder, but mechanistic insight into these reactive species is missing due to a lack of standardized methods and assessments of the redox environment. Defining validated and standardized methods to measure specific reactive species in both the basic and clinical settings is urgently needed to advance the field of redox biology in mental health. In doing so, we hope to discover new roles for these reactive species that may serve as better diagnostic or therapeutic tools for PTSD and potentially other mental health disorders.
